# Supplementary material for: Comparative Transcriptome Reveals Conserved Gene Expression in Reproductive Organs in Solanaceae
Source: Int J Mol Sci. 2025 Apr 10;26(8):3568. doi: 10.3390/ijms26083568 (PMC12027018; doi:10.3390/ijms26083568)
Supplement: Supplementary file 1 [file ijms-26-03568-s001.zip › Solanaceae ComTrans Supplementary Figures_0307.pdf]

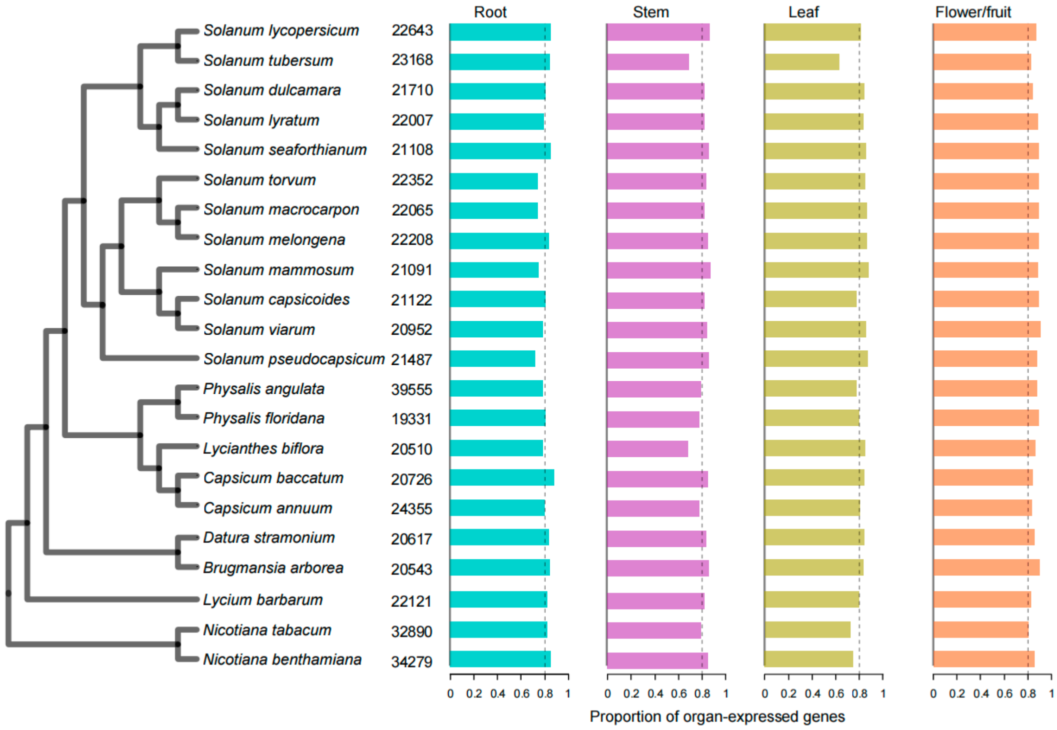

Figure S1. Characteristics of gene expression in Solanaceae. The phylogenetic tree on the left illustrates the evolutionary relationships among 22 Solanaceae species. The number following each species name represents the number of genome-wide expressed genes. The bar plot then shows the proportion of the number of genes expressed in each organ to the total number of genes expressed.

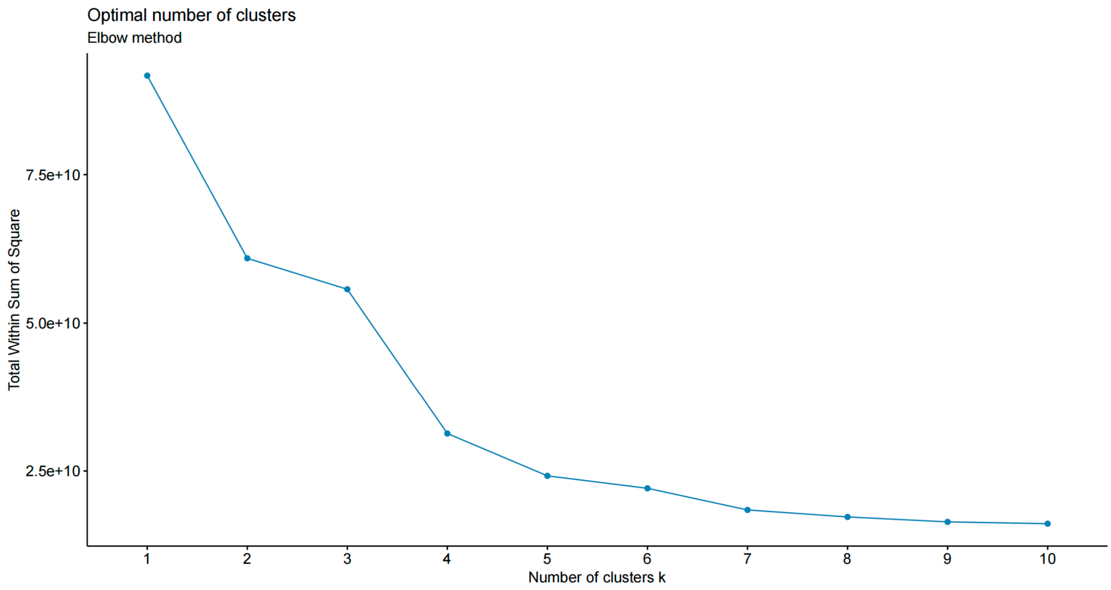

Figure S2. Optimal number of clusters for pepper and tomato temporal transcriptomes. This analysis is from ClusterGVis and when the line drops to smooth, it represents is the optimal number of clusters.

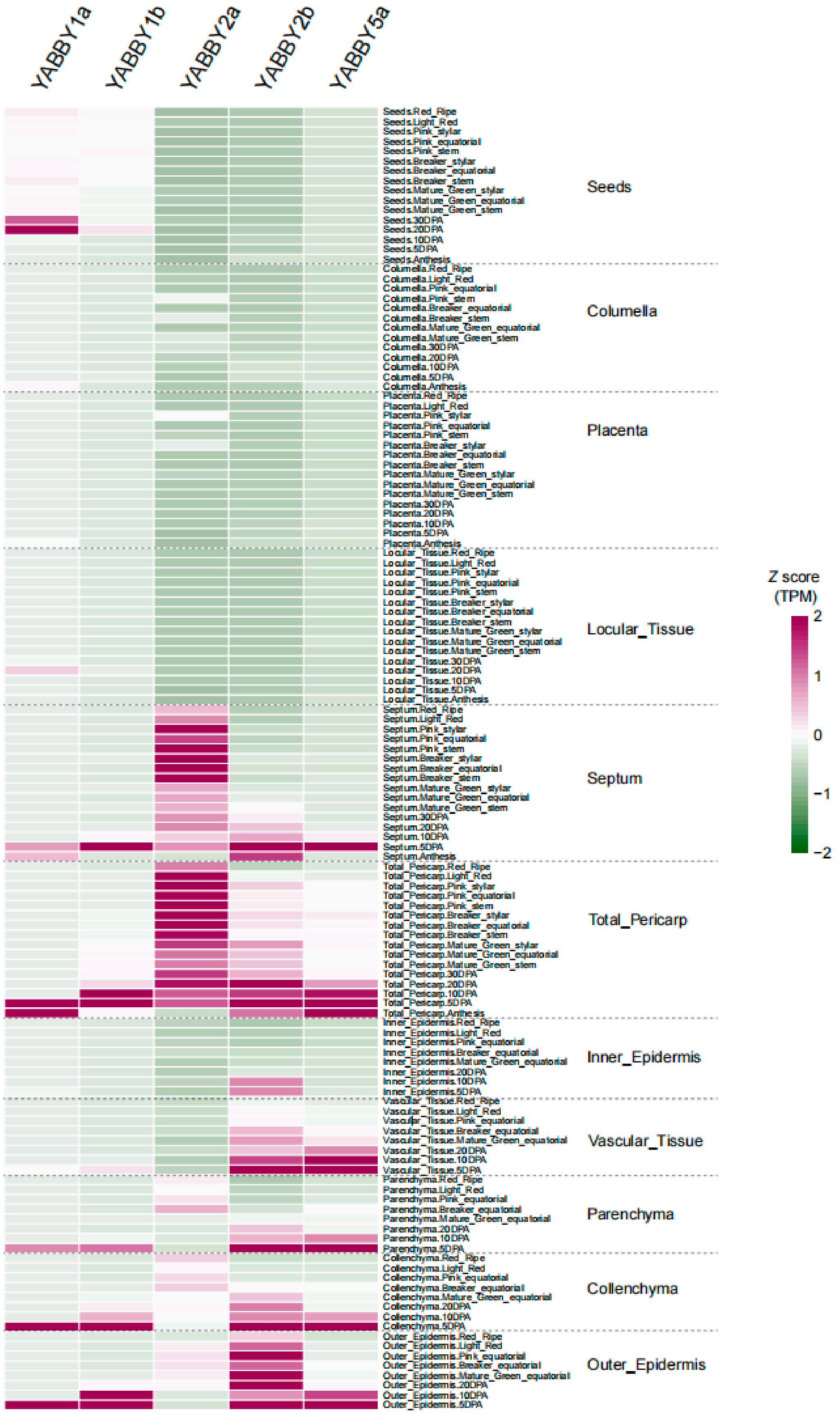

Figure S3. Heatmap displaying the expression levels of YABBY genes in tomato fruit across different tissue of different development stages.
